# Antifungal and Antivirulence Activity of Alizarin against *Candida dubliniensis*

**DOI:** 10.1021/acsinfecdis.6c00149

**Published:** 2026-06-01

**Authors:** Ganeshkumar Arumugam, Samuthirapandi Muniasamy, Patricia Michelle Nagai de Lima, Uilla Barcick, André Zelanis, Bianca Carla Silva Campitelli Barros, Lara Luise Castro Pedroso, Bruno Montanari Borges, Nycolas Willian Preite, Flavio V. Loures, Juliana Campos Junqueira

**Affiliations:** † Department of Biosciences and Oral Diagnosis, Institute of Science and Technology, São Paulo State University/UNESP, São José dos Campos, São Paulo 12245-000, Brazil; ‡ Department of Materials Physics, Saveetha School of Engineering, Saveetha Institute of Medical and Technical Sciences (SIMTS), Thandalam, Chennai, Tamil Nadu 602105, India; § Department of Microbiology, 30003 Ayya Nadar Janaki Ammal College (Autonomous, Affiliated to Madurai Kamaraj University, Madurai), Sivakasi, Tamil Nadu 626124, India; ∥ Institute of Science and Technology, 28105 Federal University of São Paulo/UNIFESP, São José dos Campos, São Paulo 12231-280, Brazil; ⊥ Laboratory of Applied Toxinology, Center of Toxins, Immune-Response and Cell Signaling, 196591 Butantan Institute, Av. Vital Brazil, São Paulo 1500-05503-000, Brazil

**Keywords:** alizarin, phytochemical, Candida dubliniensis, antifungal activity, shotgun proteomics, hyphae inhibition

## Abstract

*Candida dubliniensis* is a relatively common yet understudied fungal species present in the oral cavity of both healthy and immunocompromised individuals. Despite its increasing clinical relevance, research addressing its pathogenesis and therapeutic targeting remains limited. In this context, the present study investigated the antifungal potential of alizarin against *C. dubliniensis*. Alizarin exhibited notable antifungal activity, with a minimum inhibitory concentration (MIC) of 16 μg/mL. Checkerboard analysis further demonstrated a significant enhancement of fluconazole activity when used in combination with alizarin, indicating a synergistic interaction. In vitro antibiofilm assays demonstrated that alizarin, at its MIC level, significantly reduces both biofilm formation and preformed biofilms of *C. dubliniensis*. Notably, administration of alizarin improved the survival of *Galleria mellonella* by decreasing the fungal burden; however, its immunomodulatory potential remained lower than that of standard antifungal treatments. Furthermore, alizarin effectively inhibited filamentation, a key virulence factor, at 16 μg/mL within 2 h of exposure. After 24 h, only 2.12% of cells retained the ability to undergo hyphal transition (*p* < 0.0001). Proteomic analysis revealed significant modulation of the protein expression profile, including downregulation of proteins associated with cell wall synthesis and integrity. Overall, these findings demonstrate that alizarin exerts antifungal effects against *C. dubliniensis*, primarily by inhibiting morphological transition and modulating virulence-associated pathways. Further studies are warranted to elucidate the underlying molecular mechanisms and to explore its potential as a therapeutic agent.

## Graphical abstract

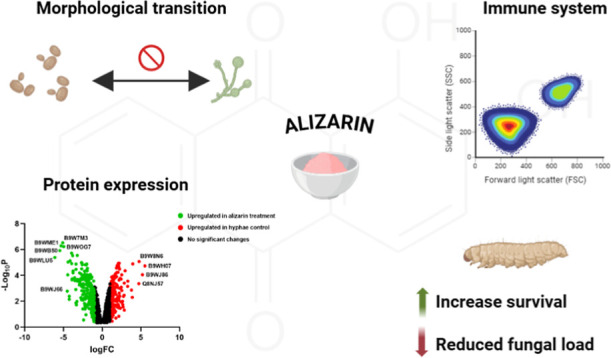

## Introduction

*Candida* is recognized as one of the most common etiological agents responsible for superficial and subcutaneous infections in immunologically impaired individuals.^1^ The genus *Candida* consists of more than 200 species; however, only a few of them are medically relevant.^2^ In particular, *Candida albicans* has been known for several decades due to its commensal behavior and colonization of different body sites in human.^3^ Colonization of *Candida* in human anatomical sites depends on several factors, including host immune response and environmental factors. Apart from the dominance of *C. albicans*, several other species have gained attention because of their medical relevance, such as *Nakaseomyces glabratus* (formerly known as *C. glabrata*), *Pichia kudriavzevii* (formerly known as *Candida krusei*), *Candida parapsilosis*, and *Candida tropicalis*. Recently, a multidrug-resistant species known as *Candidozyma auris* (formerly known as *Candida auris*) was included in this list as a critical priority fungal pathogen by the World Health Organization.^4^ Initially, *C. auris* was reported in Japanese patients presenting with otitis; however, more recently it has been detected in multiple parts of the human body, as well as in clinical and environmental samples and on various nonliving surfaces, including medical equipment and intensive care units.^5–7^ In addition to the species listed, another *Candida* species has been reported in both healthy individuals and patients with several medical conditions.

Like other nonalbicans species, *Candida dubliniensis* is widely distributed among healthy individuals.^8,9^ However, a higher prevalence has been observed in the oral microenvironment of HIV/AIDS patients. It has become an emerging opportunistic pathogen that causes superficial and systemic infections in immunocompromised individuals.^10^ *C. dubliniensis* shares most characteristic features with *C. albicans*, making differentiation difficult and leading to frequent misidentification as *C. albicans*, even with modern diagnostic pipelines.^11^ Indeed, *C. dubliniensis* was first isolated from the oral cavity of an HIV patient in 1995.^12^ Among HIV and AIDS patients with oral manifestations, *C. dubliniensis* had a recovery rate of 27% to 32% of cases.^13^ A high prevalence of this species has also been recorded in individuals presenting with other clinical manifestations, such as denture wearers,^14,15^ vulvovaginal candidiasis,^16^ diabetes,^17,18^ cystic fibrosis,^19–21^ and cancer.^22^ Surprisingly, the prevalence rate in asymptomatic immunocompromised individuals did not vary significantly, standing at 19% in those with HIV and 25% in those individuals with AIDS.^13,23^ Over the past decades, the distribution of *C. dubliniensis* in clinical settings has been continuously monitored. Recent reports provide evidence that *C. dubliniensis* can evade the immune system, causing meningitis in immunocompetent patients^24,25^ and endophthalmitis.^26,27^

Natural products such as eugenol, carvacrol, and thymol and chemically derived antiseptic agents including triclosan, chlorhexidine gluconate, and cetylpyridinium chloride have been reported to inhibit the growth of *C. dubliniensis* and other pathogenic organisms.^28^ Similarly, the plant *Capsicum annuum* ^29^ and magnetic nanoparticles^30^ have demonstrated antifungal potential against *C. dubliniensis*. While considerable attention has been given to inhibiting fungal growth, the control of morphological transitions in *C. dubliniensis* remains largely unexplored. In other *Candida* species, several compounds have been reported to target virulence factors, including biofilm formation and hyphal transition: occidiofungin,^31^ tetrachlorohydroquinone,^32^ magnolol and honokiol,^33^ 7-benzyloxyindole,^34^ and purpurin.^35^

Previous studies have shown that dihydroxyanthraquinones such as alizarin and chrysazin possess strong antifungal activity, significantly reducing hyphal formation and biofilm development in *C. albicans*.^36^ More recently, the tetrahydroxyanthraquinone derivative quinalizarin was reported to inhibit the growth of *Candida* species isolated from cancer patients.^37^ For instance, quinalizarin, an alizarin analogue with two additional hydroxyl groups, exhibits enhanced antifungal activity against common *Candida* species, including emerging pathogens like *C. auris* and other nonalbicans species that are highly resistant to fluconazole, a frontline antifungal agent used for clinical prophylaxis.^37^ Building on these findings, the present study aims to evaluate the ability of the anthraquinone derivative alizarin to control the growth and virulence properties of *C. dubliniensis* similar to those previously observed in *C. albicans*. Additionally, considering that certain phytochemicals are known to modulate host immune responses during infections, we also investigated the immunomodulatory potential of alizarin using a *Galleria mellonella* candidiasis model.

## Results and Discussion

### Alizarin Exerts Antifungal Activity through a Fungistatic Mode of Action

The antifungal activity of alizarin against *C. dubliniensis* was evaluated using the broth microdilution method. The MIC values of alizarin against clinical and ATCC strains of *C. dubliniensis*, along with *C. albicans* and other *Candida* and *Aspergillus* species, are summarized in Table 1. All tested *Candida* strains were susceptible to fluconazole (MIC = 0.5 μg/mL), except *C. krusei*, which exhibited reduced susceptibility (MIC = 32 μg/mL). In addition, all *Candida* isolates were susceptible to Amp B, with MIC values ranging from 0.25 to 0.5 μg/mL. *Aspergillus* species also demonstrated susceptibility to Amp B, with MIC values of 0.5–1.0 μg/mL. With the exception of *C. parapsilosis* and two *Aspergillus* strains (MIC > 128 μg/mL), the remaining *Candida* strains were susceptible to alizarin, exhibiting MIC values ranging from 1 to 16 μg/mL. However, alizarin displayed a MFC of 2048 μg/mL against *C. dubliniensis*. Subsequent analyses were restricted to *C. dubliniensis*, whereas other fungal species were evaluated solely to determine the broad-spectrum antifungal activity of alizarin.

**Table 1:** Minimal Inhibitory Concentration (MIC) of Alizarin and antifungals on Different Strains

S.no	organism	MIC (μg/mL)			
		Alizarin	Fluconazole	Amp B	Anidulafungin
1	C. dubliniensis—ATCC MYA-646	16	0.5	0.25	0.015
2	C. dubliniensis—P3 clinical isolate	16	0.5	0.25	0.015
3	C. albicans—ATCC SC5314	16	0.5	-	-
4	Candida krusei ATCC 6258	1	32	0.5	0.015
5	Candida parapsilosis ATCC 22019	>128	0.5	0.5	0.5
7	Aspergillus flavus ATCC 204304	>128	-	1	-
8	Aspergillus fumigatus ATCC 3626	>128	-	0.5	-

This fungistatic effect was further confirmed through time-kill kinetics studies (Figure 1), where exposure to different concentrations of alizarin (MIC, MIC × 5, MIC × 10, and MFC) reduced the viability of *C. dubliniensis*. Notably, at 2048 μg/mL, alizarin significantly decreased cell viability after 24 h of incubation. In contrast, amphotericin B (positive control) completely eradicated viable cells within 2 h, consistent with its fungicidal properties.

**Figure 1:**
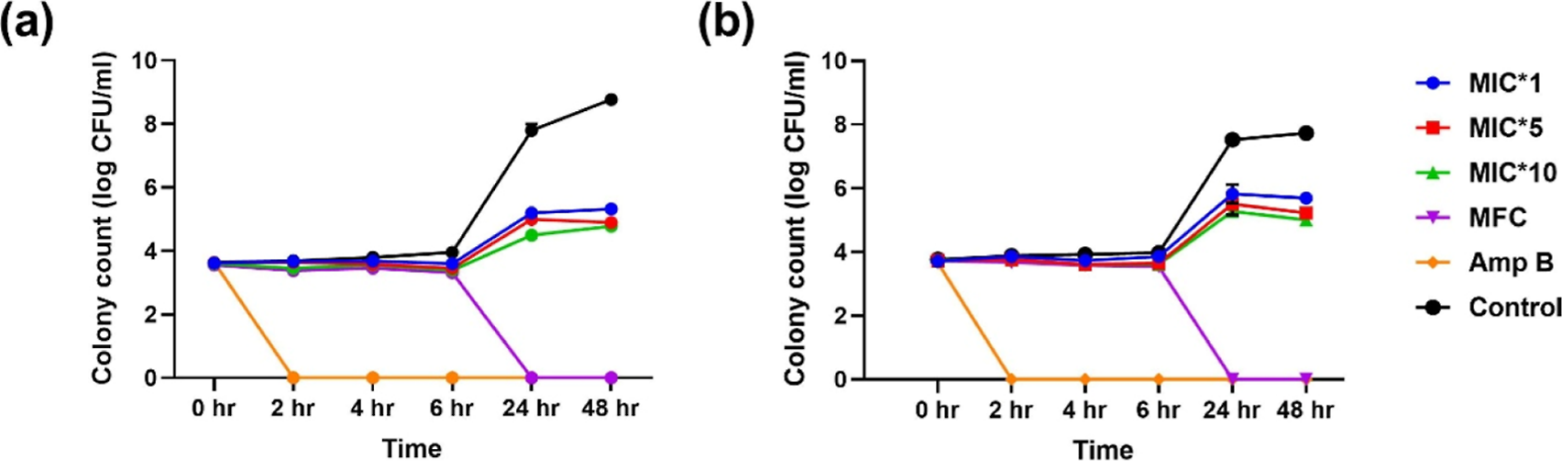
Time-kill curves were generated for *Candida dubliniensis* (CD) cells exposed to different concentrations of alizarin. Colony-forming units (CFUs) were recorded from samples collected at various time intervals for three independent samples (*n* = 3). (a) Growth pattern of the reference strain *C. dubliniensis* (ATCC MYA-646) (b) *C. dubliniensis* clinical isolate (P3). The differential responses observed in the time-kill curves confirmed that alizarin exhibited fungistatic activity, whereas amphotericin B (Amp B) demonstrated a fungicidal effect.

In the present study, we investigated the inhibitory effects of the phytochemical alizarin on the growth and key virulence traits of *C. dubliniensis*, a prevalent commensal fungus commonly detected in the oral cavity of immunocompromised patients.^38,39^ To assess the fungistatic and fungicidal properties of alizarin, the ratio between MFC and MIC was determined, where a ratio exceeding 4 indicates fungistatic activity, while a ratio below 4 suggests fungicidal action.^37,40^ Our findings demonstrated that alizarin exhibited fungistatic activity against tested *Candida* species, with an MFC value of 2048 μg/mL. This is consistent with previous reports describing the fungistatic properties of alizarin and its derivatives, such as quinalizarin, against *C. albicans* and non-*albicans* species.^36,37^ Anthraquinones are already well-documented for their antifungal activity against medically important fungi, including *Madurella mycetomatis*,^41^ pathogenic trichomonads,^42^ and diverse yeast species.^37^ In agreement with these studies, alizarin also displayed antifungal effects against the oral pathogen *C. dubliniensis*.

In previous studies, alizarin not only inhibited *C. albicans* but also suppressed the growth and metabolism of other opportunistic fungi and bacteria.^37,43^ However, in contrast to earlier investigations, we observed a strong antifungal effect of alizarin against *C. dubliniensis*, with MIC values several fold lower than those reported for *C. albicans*. For example, Manoharan et al. (2017) reported MIC values exceeding 2000 μg/mL for alizarin against *C. albicans*.^36^ In our study, however, alizarin exhibited MIC values of 16 μg/mL against *C. dubliniensis* (ATCC MYA-646) and *C. albicans* (ATCC SC5314). This variability may reflect differences in strain-specific susceptibility and resistance behavior. The strains we tested were highly sensitive to fluconazole (MIC = 0.5 μg/mL; Table 1), whereas the strains used by Manoharan et al. (2017) may have been resistant,^36^ which could account for their elevated MIC values. Interestingly, this discrepancy was observed only for alizarin, while quinalizarin a structurally related derivative showed consistently lower MIC values across *Candida* strains, including those resistant to fluconazole, underscoring how minor structural modifications can enhance antifungal activity.^37^

### Effect of Alizarin on a *C. dubliniensis* Biofilm

The antibiofilm activity of alizarin was evaluated against both ATCC and P3 strains of *C. dubliniensis* and is presented in Figure 2. At 16 μg/mL (MIC), alizarin affected the biofilm-forming ability of both strains. At this concentration, biofilm formation was reduced by 22% and 33% in the ATCC and P3 strains, respectively. With increasing concentrations, a dose-dependent effect was observed. Notably, at MIC × 10 (160 μg/mL), biofilm biomass was reduced by 49.01% in the ATCC strain and 48.03% in the P3 strain. Further analysis demonstrated that alizarin also exhibited biofilm eradication potential. At MIC (16 μg/mL), only 16.77% and 24.23% of preformed biofilms were eradicated in the ATCC and P3 strains, respectively. However, higher concentrations significantly enhanced biofilm reduction, with biomass decreases of 35.70% (ATCC) and 39.89% (P3). These results indicate that alizarin has the potential to inhibit biofilm formation in both strains of *C. dubliniensis*. However, the concentration required to achieve approximately 50% reduction in biofilm biomass is relatively high compared to typical antifungal activity thresholds. Therefore, further toxicological studies are necessary to evaluate the pharmacological potential and safety profile of alizarin.

**Figure 2:**
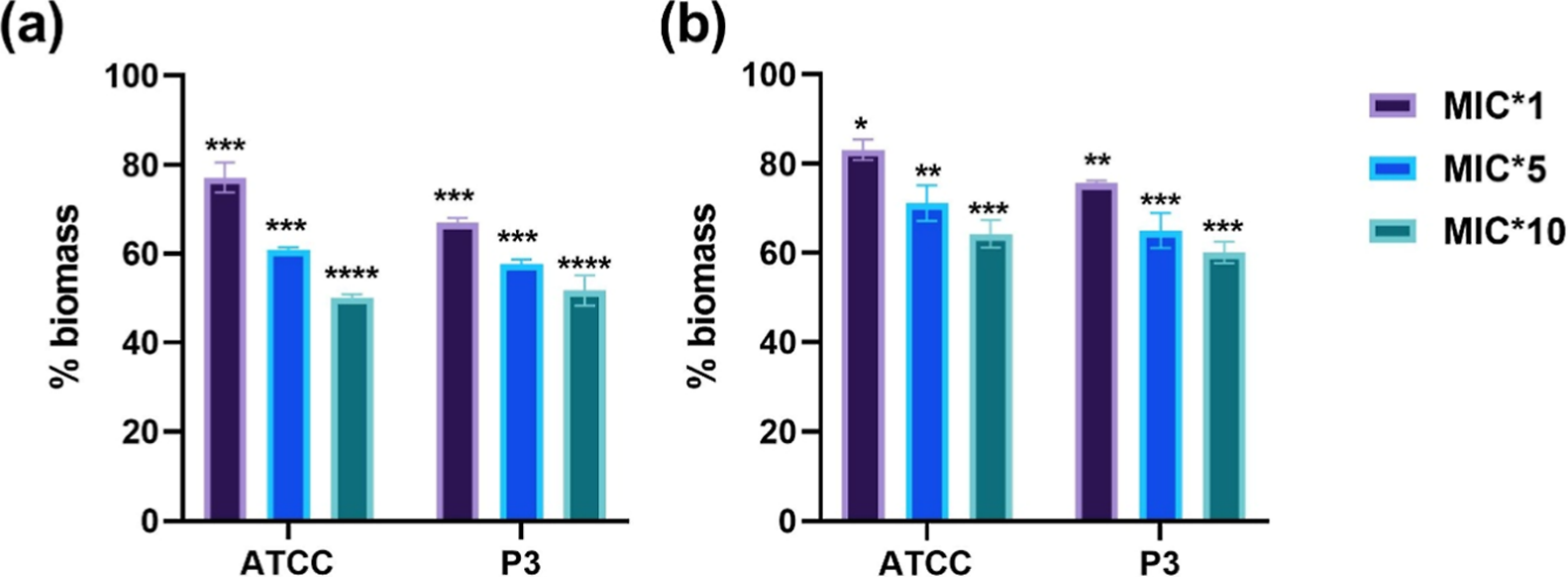
Antibiofilm activity of alizarin against biofilm formation (a) and preformed biofilms (b) at different concentrations. Statistical significance was determined using one-way ANOVA. Significance levels are indicated as * (*p* = 0.01), ** (*p* = 0.001), *** (*p* = 0.0001), and **** (*p* < 0.0001).

In the present study, alizarin exerted a stronger inhibitory effect on biofilm formation (up to 49.90%) than on preformed biofilms (39.89%) of *C. dubliniensis*. This suggests that alizarin is more effective during the early stages of biofilm development rather than against mature biofilms, indicating a stage-specific activity. This observation is consistent with the well-established resistance mechanisms of mature biofilms, which are enriched with an extracellular matrix that restricts antifungal penetration and limits the direct interaction with fungal cells. The antibiofilm activity of alizarin has previously been reported in *C. albicans*, where it inhibits biofilm formation and downregulates key virulence-associated genes.^36^ Similarly, related anthraquinones, including chrysazin and purpurin, exhibit moderate antibiofilm effects, while quinalizarin demonstrates strain-dependent variability in its activity.^37^ Although gene expression analysis was not performed in the present study, the observed reduction in biofilm biomass suggests that alizarin may act through similar mechanisms in *C. dubliniensis*. Furthermore, alizarin has been reported to inhibit both mono- and multispecies biofilms, indicating a broader antimicrobial potential.^43^

Most anthraquinone derivatives reported to date exhibit predominantly fungistatic rather than fungicidal activity against pathogenic fungi, suggesting their unique mode of action. Importantly, these compounds often display significant antivirulence properties, including inhibition of biofilm formation and morphological transition, frequently through modulation of the cellular energy metabolism in *C. albicans*. For instance, 2-chloromethyl anthraquinone has been shown to significantly inhibit biofilm formation in *C. albicans* by targeting the Ras1-cAMP-Efg1 signaling pathway without affecting planktonic growth.^44^ Similarly, parietin, a natural anthraquinone, disrupts *C. tropicalis* biofilms via photoinactivation.^45^ Photodynamic approaches using anthraquinones are also gaining attention; for example, aloe-emodin has been reported to effectively control biofilms of both azole-sensitive and resistant *C. albicans* under light irradiation.^46^ In line with this, previous studies have demonstrated that quinone derivatives can suppress virulence traits in *C. albicans* without markedly affecting fungal growth.^47^ Additionally, 2-ethylanthraquinone has been shown to primarily target the biofilm matrix rather than planktonic yeast cells.^48^ Moreover, these findings indicate that anthraquinones and their derivatives are characterized by strong antibiofilm and antivirulence activities, even in the absence of potent fungicidal effects. Such properties highlight their potential as alternative or adjunct therapeutic strategies for managing fungal infections, similar to those of clinically used fungistatic agents such as fluconazole.

### Alizarin Can Modulate the Antifungal Activity of Fluconazole

The antifungal activity of alizarin, alone and in combination with standard antifungals such as fluconazole, amphotericin B, and anidulafungin, was evaluated using a checkerboard microdilution assay. Alizarin alone exhibited an MIC of 16 μg/mL; however, when combined with antifungal agents, a reduction in MIC values was observed. Combinations with amphotericin B or anidulafungin resulted in a onefold dilution decrease in MIC, although FICI analysis indicated an indifferent interaction. In contrast, the combination of alizarin with fluconazole demonstrated a pronounced synergistic effect. In both tested strains, the combination of 4 μg/mL alizarin and 0.004 μg/mL fluconazole yielded a FICI value ≤ 0.5, confirming synergy (Table 2). These findings suggest that the alizarin-fluconazole combination may enhance antifungal efficacy against *C. dubliniensis* and represents a promising combinatorial strategy (see Table 3).

**Table 2:** Interaction between Alizarin (Drug A) and Fluconazole (Drug B) against *C. dubliniensis* Strains^a^

strain	MIC of drug A	MIC of drug B	FIC A	FIC B	FICI	effect
ATCC	16	0.5	1	0.008	1.008	additive
	16	0.5	0.5	0.008	0.508	additive
	16	0.5	0.25	0.008	0.258	synergistic
P3	16	0.5	1	0.008	1.008	additive
	16	0.5	0.25	0.008	0.258	synergistic
	16	0.5	1	0.004	1.004	additive

a Only concentrations exhibiting inhibitory activity were included.

**Table 3:** List Upregulated Proteins in the Untreated Group

function of protein	log fold change	gene name	protein name
energy metabolism	1.35	RGI1 CD36_22420	respiratory growth induced protein 1
	1.42	CD36_50250	glucosidase II subunit alpha
	1.07	PMI1 CD36_23720	mannose-6-phosphate isomerase (EC 5.3.1.8)
	1.23	NTC1 CD36_25660	trehalase (EC 3.2.1.28) (alpha-trehalose glucohydrolase)
	1.37	CD36_30070	UTP-glucose-1-phosphate uridylyltransferase (EC 2.7.7.9)
	1.64	CD36_42770	glycosidase (EC 3.2.-.-)
	1.13	CD36_87400	phosphoglucomutase, putative (EC 5.4.2.2)
	1.74	CD36_34550	HMG-CoA synthase (EC 2.3.3.10)
	1.42	CD36_09120	sphingolipid C(9)-methyltransferase (EC 2.1.1.317)
	2.52	CD36_16240	lysophospholipase (EC 3.1.1.5)
cell wall	4.77	CD36_08100	squalene monooxygenase (EC 1.14.14.17)
	1.39	CD36_81470	glucan endo-1,3-beta-d-glucosidase (EC 3.2.1.39)
	2.44	CD36_72510	sterol 22-desaturase (EC 1.14.19.41)
	2.85	ERG6 CD36_82130	sterol 24-C-methyltransferase (EC 2.1.1.41)
	1.30	KRE9 CD36_84120	cell wall synthesis protein, putative
	3.78	CHT2 CD36_53830	chitinase (EC 3.2.1.14)
	2.09	CYB55 CD36_70650	cytochrome B5, putative
	3.29	CD36_52240	uncharacterized GPI-anchored cell wall protein, putative
	1.84	CD36_61820	integral membrane protein, putative
	4.74	ERG3 CD36_04520	(C-5 sterol desaturase)
	5.51	ERG11 CD36_50660	lanosterol 14-alpha demethylase
oxidative stress	1.92	CD36_00330	cell survival pathways effector, SVF-family, putative
	1.06	CD36_24320	calcium/calmodulin-dependent protein kinase, putative
filamentation	1.21	CD36_84850	SH3 domain-containing protein, putative
	2.24	HGT6 CD36_15940	hexose transporter, putative
	2.04	CSH3 CD36_43160	endoplasmic reticulum packaging chaperone, putative
	3.31	PHR1 CD36_44230	1,3-beta-glucanosyltransferase (EC 2.4.1.-)
	1.17	PHR2 CD36_00220	1,3-beta-glucanosyltransferase (EC 2.4.1.-)
	1.76	UTR4 CD36_16690	enolase-phosphatase E1 (EC 3.1.3.77)

### Alizarin Is Nontoxic, Reduces the Fungal Burden in *G. mellonella*, and Improves Its Survival against *C. dubliniensis*

The toxicity of alizarin was assessed using the *G. mellonella* model. Larvae were administered different concentrations of alizarin (16 and 32 μg/larva), and survival rates were monitored over a period of 5 days. No significant mortality was observed at either concentration, indicating the nontoxic nature of alizarin, whereas DMSO showed 100% mortality at the first day postinjection (Figure 3a). Given the antifungal activity exhibited by alizarin in vitro, its in vivo efficacy was further evaluated using the *G. mellonella* candidiasis infection model. The survival curves for both the control and PBS groups showed 100% survival throughout the experimental period for both P3 and ATCC strains, confirming that handling procedures and vehicle administration did not contribute to larval mortality and thereby validating the infection model. In contrast, the disease control groups exhibited a progressive decline in survival, demonstrating that fungal infection caused significant mortality in *G. mellonella*. Mortality was more pronounced in the P3-infected larvae compared to the ATCC group, consistent with the greater virulence of the P3 strain, whereas Amp B treatment consistently improved survival in infected larvae. As shown in Figure 3b,c, immediate administration of alizarin conferred only partial protection, while treatment 2 h prior to infection provided limited benefit, with survival curves closely overlapping those of the disease control group. Notably, administration of alizarin 24 h prior to infection resulted in the greatest improvement in larval survival, indicating a time-dependent prophylactic effect. Consistent with these observations, Mantel-Cox (log-rank) analysis demonstrated significant differences among survival curves for both P3 (*p* = 0.0035) and ATCC (*p* = 0.0281) infections, supporting the conclusion that alizarin treatment significantly improves disease outcomes in the *G. mellonella* infection model.

**Figure 3:**
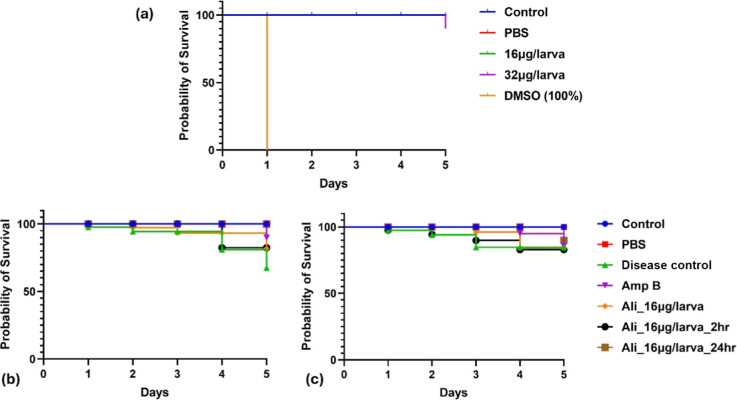
(a) Toxicity assessment of alizarin in vivo using the *Galleria mellonella* infection model. Differential doses of alizarin were administered to evaluate its antifungal potential in *G. mellonella* larvae infected with (b) *C. dubliniensis* ATCC MYA-646 and (c) the *C. dubliniensis* clinical isolate (P3). Survival analysis using the Mantel-Cox test revealed significant differences among the treatment groups (*p* = 0.028 for the ATCC strain and *p* = 0.0035 for the P3 clinical isolate). (PBS—phosphate buffer saline; DC—disease control; Ali MIC—alizarin injected 30 min after infection; Ali_MIC_2 h—injected 2 h after infection; Ali_MIC_24 h—alizarin injected 24 h prior to infection).

After 5 days of treatment, the hemolymph was aseptically collected from larvae in each group using the sterile method described in the Materials and Methods section. Fungal burden analysis revealed no viable fungal colonies in control and PBS-administered larvae. However, a significant number of colonies were observed in the disease control group (9.16 CFU/10 μL for the ATCC strain and 11 CFU/10 μL for the P3 strain). The higher fungal burden in the P3-infected group suggests greater virulence of this strain compared to the ATCC strain (Figure 4). Further analysis demonstrated that both alizarin and amphotericin B (Amp B) injections reduced fungal colony counts at 30 and 120 min post-treatment. Notably, larvae pretreated with alizarin 24 h prior to infection exhibited a significant reduction in viable fungal colonies compared with the disease control group (*p* = 0.008 for ATCC; *p* = 0.0038 for P3), highlighting its protective effect against *Candida* infection.

**Figure 4:**
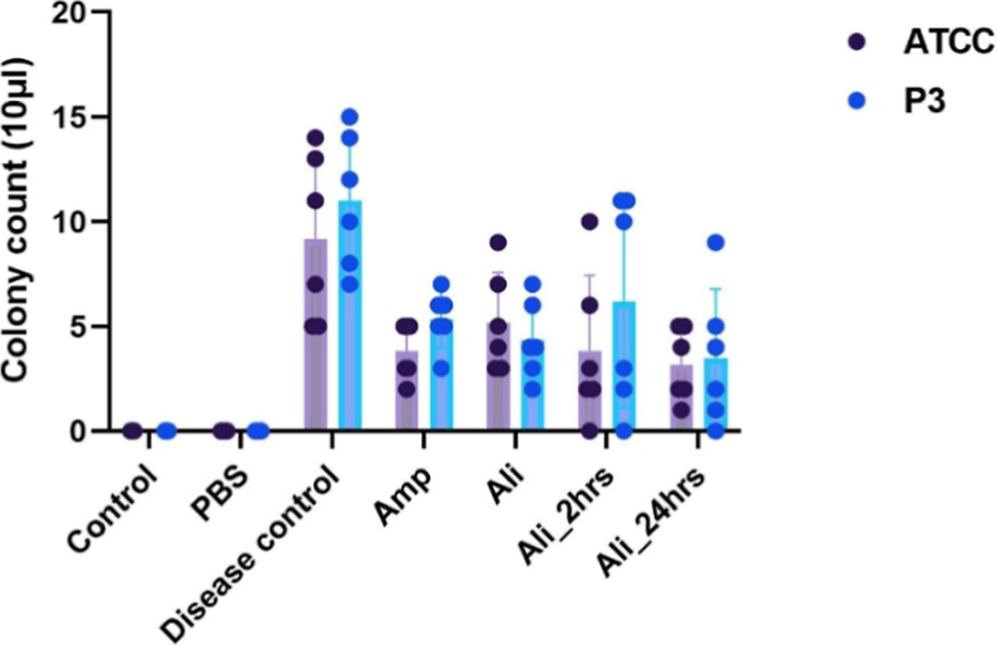
Fungal burden analysis of *G. mellonella* larvae treated with varying concentrations of alizarin and amphotericin B. Compared to the disease control, both alizarin and amphotericin B significantly reduced fungal cell counts in larvae infected with *C. dubliniensis* ATCC MYA-646 and (b) the *C. dubliniensis* clinical isolate (P3). Mean values of three independent samples (*n* = 3) have been presented.

Anthraquinones, including alizarin, are generally associated with low toxic potential, although their safety can vary depending on functional group modifications. In our study, alizarin demonstrated no detectable toxicity in the *G. mellonella* model at concentrations up to 32 μg/larva. Similarly, quinalizarin exhibited minimal toxicity in human erythrocytes, causing only 2% hemolysis at MIC levels, though higher concentrations (40–160 μg/mL) resulted in ∼20% hemolysis.^37^ Another study reported that alizarin was well-tolerated by *Caenorhabditis elegans* at concentrations up to 1 mg/mL, further supporting its biocompatibility (Manoharan et al., 2017). These findings are consistent with our results and those of previous investigations.^37^

Different disease models have been widely employed to evaluate the therapeutic potential of phytochemicals, with *G. mellonella* and *C. elegans* candidiasis models being particularly useful.^49,50^ In our study, *G. mellonella* larvae infected with *C. dubliniensis* showed significantly reduced survival, while treatment with alizarin significantly improved survival rates, confirming its antifungal efficacy in vivo. These findings are in line with previous work showing that alizarin extended the lifespan of *C. elegans* infected with *C. albicans*, with 60% of nematodes surviving 4 days postinfection.^36^ Collectively, improved survival in infected host models correlates strongly with the antifungal effect of Alizarin.

### Treatment with Alizarin Showed Significant Variations in Immune Cells of *G. mellonella*

Immunophenotyping of hemocytes from *G. mellonella* infected with *Candida* provides insights into the immune modulation induced by alizarin treatment. Following differential treatment with alizarin, hemocytes were aseptically collected and analyzed by flow cytometry on the basis of size and granularity. The comparative analysis of hemocyte populations revealed distinct immune responses among different treatment groups. Infection with *C. dubliniensis* (DC) significantly reduced the prohemocyte (P1) and plasmatocyte (P2) populations, while it increased granulocyte (P3), spherule cell (P5), and oenocytoid (P7) levels, reflecting an active but strained immune response (Figure 5). Alizarin treatment partially restored P1 levels (1.18-fold) and moderately reduced P3 populations (0.68-fold) compared with the disease control, suggesting a partial immune activation and pathogen clearance. However, the P1 (1.81-fold) recovery and P3 reduction (0.37-fold) were not as pronounced as those observed with amphotericin B, which brought hemocyte distributions closer to baseline levels seen in healthy controls. Notably, spherule cells (P5) remained elevated with alizarin exposure (1.06-fold), indicating ongoing immune stimulation, whereas Amphotericin B more effectively normalized their levels (0.93-fold). These findings suggest that while alizarin can enhance immune responses against fungal infections, its efficacy remains lower than that of the standard antifungal treatment.

**Figure 5:**
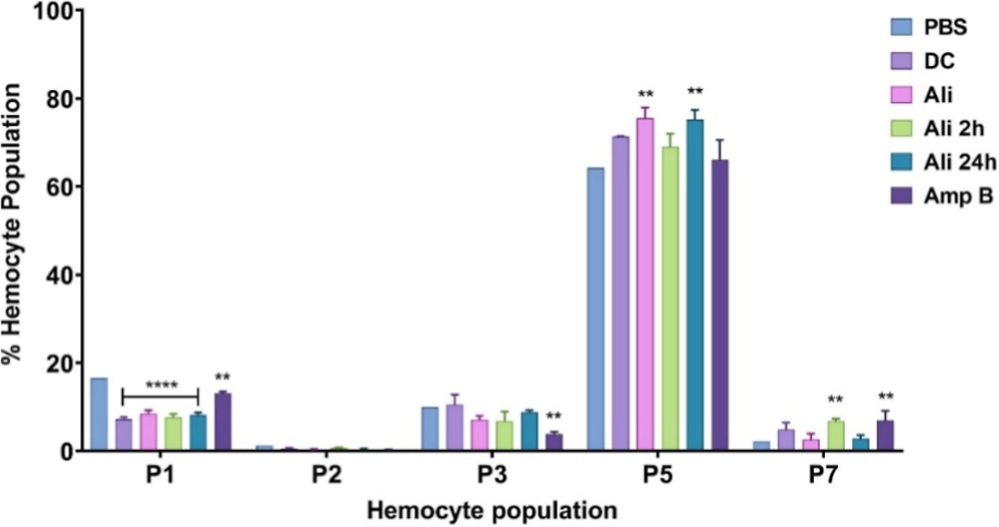
FACS analysis of *Galleria mellonella* hemocytes based on size and granularity. Five distinct hemocyte populations were identified, with P5 being the most dominant, followed by P1 and P3. The proportion of P2 cells was comparatively lower than the other populations. Significant variations were observed in P1, P5, and P7 following alizarin exposure compared to control groups, suggesting that alizarin modulates the immune response of *Galleria mellonella*. However, this immune activation appears insufficient to achieve superior immune modulation. (**p = 0.001; ****p < 0.0001) (PBS—phosphate buffer saline; DC—disease control; Ali—Alizarin).

### Alizarin Potentiates Morphological Transition in *C. dubliniensis*

Given that alizarin is known to inhibit hyphal formation in *C. albicans*, we investigated its effect on the morphological transition of *C. dubliniensis*, a key virulence determinant. Prior to these experiments, both clinical and ATCC strains were assessed for their ability to undergo morphological transition. The clinical isolate exhibited a higher propensity for hyphal formation than the ATCC strain and was therefore selected for further analysis. In the presence of 10% fetal bovine serum (FBS), cells initiated hyphal development. However, the addition of 16 μg/mL alizarin significantly inhibited hyphal formation from 2 h of incubation onward, with only 6.44% of cells exhibiting germ tubes (the structure that initiate the transition from yeast to hyphae in *Candida* species) (*p* < 0.0001), compared to 66.24% in the control group (Figure 6). When alizarin concentration was reduced by onefold or 4-fold, the proportion of hyphae-positive cells increased to 30.74% (*p* < 0.0001) and 54.15% (*p* = 0.0651), respectively. After 24 h of exposure, only 2.12% of cells retained the ability to transition into hyphal forms (*p* < 0.0001), confirming that alizarin effectively modulates the morphological transition of *C. dubliniensis*, similar to its effect on *C. albicans* (Figure 7).

**Figure 6:**
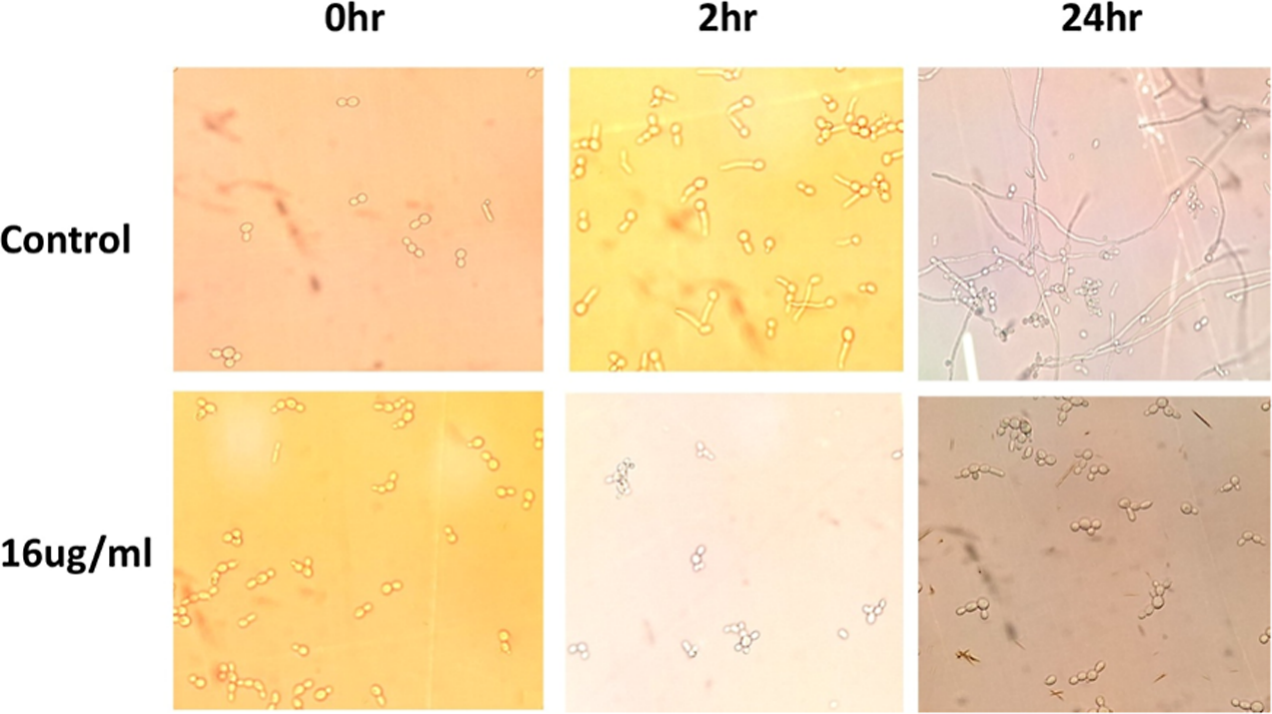
Hyphae inhibition by alizarin in *Candida dubliniensis* (P3 clinical isolate). Treatment with 16 μg/mL of alizarin for 2 h effectively suppressed the morphological transition to hyphal forms.

**Figure 7:**
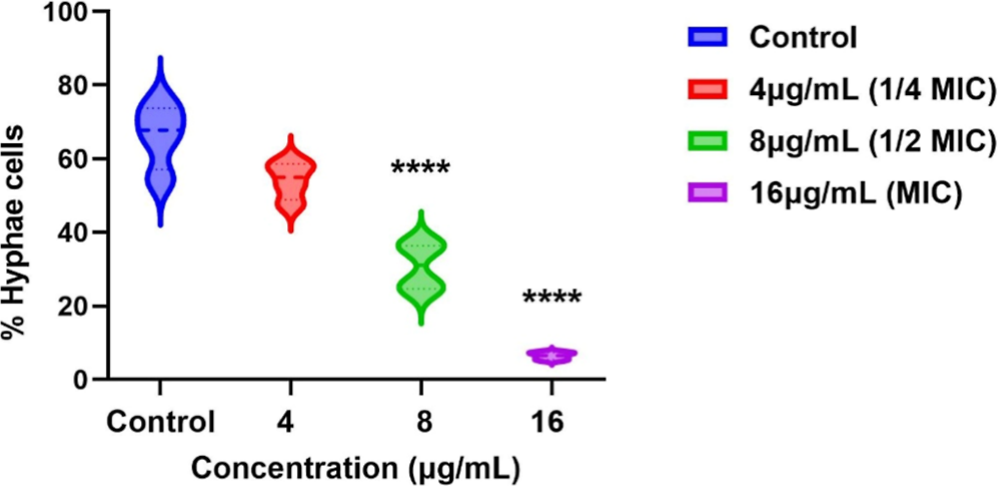
Dose-dependent inhibition of hyphae formation by alizarin in the presence of 10% FBS. Experiments were performed at three independent time points (*n* = 3). As the concentration of alizarin decreased, the rate of hyphal formation gradually increased. (****p < 0.0001).

The antifungal properties of alizarin and related anthraquinones are well-established against *C. albicans*, where they inhibit morphological transition, disrupt biofilm formation, and downregulate virulence-related genes in a dose-dependent manner.^36^ In line with these observations, alizarin at its MIC (16 μg/mL) effectively blocked the yeast-to-hyphae transition in *C. dubliniensis*, a critical determinant of pathogenicity. However, at sub-MIC concentrations (1/2 MIC and 1/4 MIC), hyphae formation significantly increased compared with untreated controls. Notably, the hyphae-transition inhibitory concentration of alizarin in *C. dubliniensis* (16 μg/mL) was several folds higher than that required for *C. albicans* (2 μg/mL).^36^

### Alizarin Exposure Modulates the Proteomic Profile of *C. dubliniensis*

To identify proteins with differential abundance in *C. dubliniensis* in response to alizarin treatment, the proteomic profile of the fungal pathogen was evaluated by LC–MS/MS. Three biological replicates were performed, and proteins detected in at least two replicates were considered for further analysis. The data were highly reproducible, with an average Pearson correlation coefficient (R^2^) of 0.976 for alizarin-treated samples and 0.995 for controls. A total of 1695 proteins were identified, of which 1431 were common to both untreated and treated samples. Additionally, 115 proteins were exclusive to untreated samples, while 149 were unique to alizarin-treated samples (Figure 8a, Venn diagram). Volcano plot analysis was used to identify proteins with differential abundance (Figure 8b). On the basis of statistical significance, proteins with a fold change greater than 1 were considered for further evaluation by comparing alizarin-treated cells with unexposed *Candida* cells. Among 1461 proteins detected, 172 proteins were increased upon alizarin treatment, while 145 were increased in the control group. These proteins were functionally classified by using UniProt ID mapping and ShinyGO analysis. A subset of functionally relevant proteins is listed in Table 4.

**Figure 8:**
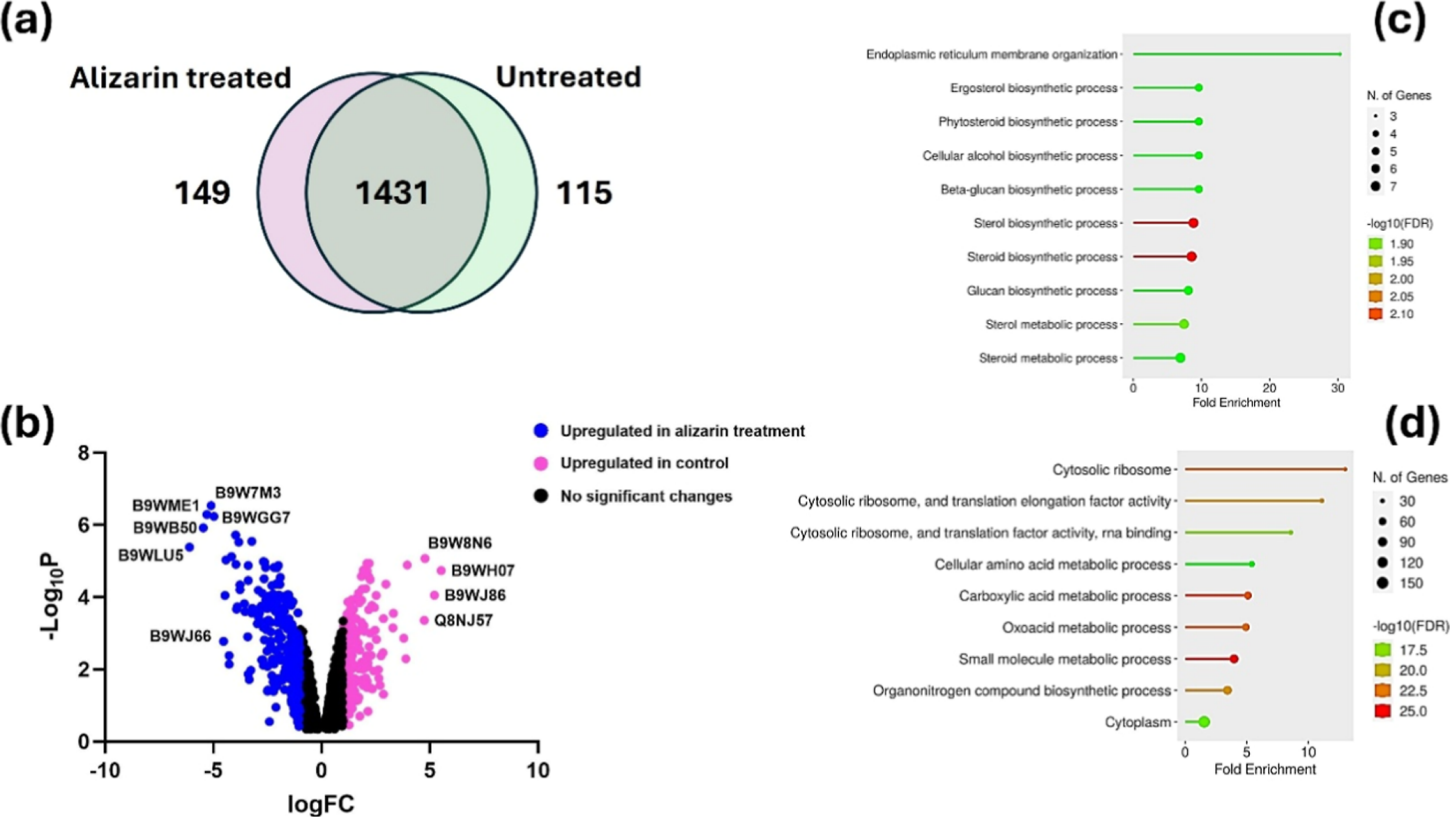
Proteomic profile analysis for alizarin-exposed and untreated *C. dubliniensis* cells. (a) The Venn diagram depicts the common and exclusive proteins associated with alizarin treatment, identifying 1431 shared proteins. (b) A volcano plot visualizes the differential abundance of these proteins upon alizarin exposure in comparison to untreated cells. Proteins with a minimum 1-fold change in expression, based on statistical significance, are highlighted in different colors, while proteins shown in black indicate no statistically significant change in relative abundance. (c,d) represent the gene ontology analysis for the control and alizarin-treated groups, respectively. Graphs are presented as the mean of three independent samples (*n* = 3).

**Table 4:** List of Upregulated Proteins in the Alizarin-Treated Group

function of protein	log fold change	gene names	protein names
amino acid metabolism	1.05	CD36_04870	phospho-2-dehydro-3-deoxyheptonate aldolase (EC 2.5.1.54)
	2.70	HIS4 CD36_40210	histidine biosynthesis trifunctional protein
	1.10	CD36_41790	malate dehydrogenase (EC 1.1.1.37)
	2.14	CD36_44080	homoaconitase, mitochondrial (EC 4.2.1.36)
	1.52	CaPDC11 CD36_46050	pyruvate decarboxylase, putative
	1.50	CD36_73220	arginosuccinase
	2.27	CD36_72980	serine hydroxymethyltransferase (EC 2.1.2.1)
	2.58	LEU1 CD36_25490	3-isopropylmalate dehydratase (EC 4.2.1.33)
	1.05	CD36_25650	malate dehydrogenase (EC 1.1.1.37)
	3.90	INO1 CD36_35120	inositol-3-phosphate synthase (EC 5.5.1.4)
	2.77	CD36_11440	multifunctional tryptophan biosynthesis protein (EC 4.1.1.48)
energy production	5.11	CD36_04240	isocitrate lyase
	2.30	GFA1 CD36_82250	glutamine--fructose-6-phosphate transaminase
	1.30	CD36_50290	ribose-phosphate diphosphokinase (EC 2.7.6.1)
	2.21	PCK1 CD36_25230	phosphoenolpyruvate carboxykinase (ATP) (EC 4.1.1.49)
	2.73	CD36_31190	aconitate hydratase, mitochondrial (Aconitase) (EC 4.2.1.-)
	3.93	CD36_35150	NADH-ubiquinone oxidoreductase subunit putative (EC 1.6.5.3)
protein production and processing	2.53	CD36_02320	60S ribosomal protein L10, putative
	1.06	CD36_05400	60S ribosomal protein L7, putative
	2.67	CD36_15190	ribosomal protein L38E, putative
	2.24	CD36_18490	40s ribosomal protein S9–B, putative
	2.55	CD36_81410	40S ribosomal protein S7
	2.30	CD36_84550	RNA-binding RNA annealing protein, putative
	1.02	CD36_85230	60S ribosomal protein L8
	2.21	CD36_86250	presequence protease
	1.21	CD36_71100	part of 40S ribosomal subunit, putative
	1.22	CD36_71620	large subunit ribosomal protein, putative
	2.72	CD36_33250	40S ribosomal protein S27
cellular respiration	1.99	CD36_00520	elongation factor Tu
	2.52	CD36_09080	adenylosuccinate synthetase (EC 6.3.4.4)
	1.20	CD36_19470	quinol-cytochrome-c reductase (EC 7.1.1.8)
	1.10	CD36_84370	cytochrome b-c1 complex subunit (EC 7.1.1.8)
	1.41	CD36_40330	ATP synthase subunit beta (EC 7.1.2.2)
	1.23	CD36_54830	ubiquinol-cytochrome-c reductase complex, putative
	1.03	CD36_63580	cytochrome b-c1 complex subunit 7
oxidative stress	3.23	SOD3 CD36_70320	superoxide dismutase (EC 1.15.1.1)
	2.75	ACT1 CD36_12690	actin (EC 3.6.4.-)
	3.53	POX1 CD36_81950	acyl-coenzyme A oxidase

Most of the proteins that increased in alizarin-treated groups are primarily involved in energy production, cellular respiration, amino acid metabolism (HIS4, LEU1, and INO1), and protein production and processing. This observation is further supported by gene ontology (GO) analysis (Figure 8c,d). GO terms analysis revealed that in the control group, most identified GO terms were associated with sterol metabolism and cell wall-related components. Among these, the sterol and steroid biosynthetic processes were significantly enriched with the highest number of genes involved (Figure 8c). However, genes involved in endoplasmic reticulum membrane organization were less represented compared with other processes. Under alizarin treatment, the most enriched GO terms were related to small-molecule metabolic processes, followed by cellular amino acid and organic nitrogen compound biosynthetic processes (Figure 8d).

Suppression of hyphal development under hypha-transition inducing conditions in *Candida* activates the general amino acid control pathway, leading to the upregulation of genes involved in amino acid biosynthesis. Metabolomic analyses of *C. albicans* isolated from various human anatomical sites have shown significant variations in intra- and extracellular amino acid levels.^51^ Notably, fluctuations in amino acids such as tryptophan, methionine, leucine, and alanine are strongly correlated not only with the organism’s primary metabolism but also with its virulence behavior.^52^ Additionally, histidine and leucine concentrations were significantly elevated after 6 h of farnesol exposure.^53^ Similarly, the addition of amphotericin B to *C. albicans* growth medium increased leucine production by 1.78-fold.^54^ In the present study, after 24 h of exposure, *Candida* cells exhibited enhanced biosynthesis of histidine (2.69-fold upregulation) and leucine (2.58-fold upregulation). Furthermore, the protein (INO1) involved in the inositol biosynthetic process, showed increased abundance by 3.8-fold following alizarin treatment in *C. dubliniensis* and in *C. albicans* a 3.67-fold decrease when exposed to amphotericin B.^54^ These variations in the inositol biosynthetic pathway may be attributed to the distinct mechanisms of action of the compounds. Alizarin appears to inhibit the growth of *C. dubliniensis* indirectly, without exerting a noticeable effect on inositol metabolism. In contrast, amphotericin B directly targets the cell membrane of *C. albicans*, leading to disruptions in several essential metabolic pathways, including those involved in inositol biosynthesis. This disturbance may result from increased oxidative stress and subsequent collapse of cellular processes.

Proteins involved in energy metabolism and cellular respiration were significantly increased in the alizarin-treated group compared to the untreated control group. This result contrasts with Han et al.’s findings (2012), who reported that after 6 h of farnesol exposure, *C. albicans* exhibited upregulation of pathways related to central carbon and energy metabolism.^53^ Similarly, *C. albicans* cells exposed to amphotericin B showed increased levels of metabolites such as malate, citrate, and oxoglutarate, all of which involved in the TCA cycle.^54^ Since alizarin inhibits the morphological transition of *C. dubliniensis*, cellular respiration levels remained higher. Additionally, alizarin influenced the cellular phase regulation of *Candida*, as evidenced by the increased abundance of α-trehalose glycohydrolase in the control group (1.23-fold). Trehalose, a minimally reactive sugar, serves as both an energy reservoir and a protectant against oxidative stress in a biological system. It is catabolized by α-trehalose glycohydrolase, whose activity typically peaks during the exponential growth phase of metabolically normal *Candida* cells.^55^

Most notably, proteins involved in energy metabolism and fermentation showed an increased abundance upon alizarin exposure. The increase of alcohol dehydrogenase confirms active fermentation in treated cells. Meanwhile, the elevated levels of glyoxylate cycle enzymes and formate dehydrogenase indicate enhanced respiratory metabolism. These findings suggest that under hypha transition inhibiting conditions, *C. dubliniensis* undergoes a significant metabolic shift, leading to an increase in fermentation- and respiration-related enzymes. This response may represent a stress adaptation or a survival strategy. In contrast, *C. albicans* exhibits higher alcohol dehydrogenase levels in hyphal cells compared to yeast cells, while proteins associated with respiration, such as Atp2 and Rib3, are decreased.^56^

Superoxide dismutase 3 (SOD3) levels increased by 3.2-fold in the alizarin-treated group, indicating that *C. dubliniensis* cells adapt to counteract free radicals generated during the morphological transition. Unlike other SODs, SOD3 uniquely functions as a manganese-containing superoxide dismutase, playing a crucial role in protecting against oxidative stress, particularly during the stationary phase when nutrients are limited.^57^ Moreover, the elevated levels of SOD3 in the alizarin-treated group compared with the control suggest a protective cellular response to oxidative stress induced by alizarin exposure.

In contrast, proteins associated with cell wall synthesis and maintenance of cell wall integrity were increased in the control group. Specifically, these proteins are involved in sterol biosynthesis, sterol metabolism, ergosterol biosynthesis, β-glucan biosynthesis, lipid biosynthesis, and polysaccharide metabolism. Notably, four key genes ERG11 (lanosterol 14-alpha-demethylase), ERG3 (C-5 sterol desaturase), PHR1, and PHR2 (putative cell wall proteins) were consistently identified across gene ontology (GO) functional enrichment analysis. The formation and elongation of hyphae require continuous cell wall remodeling, which drives the increased production of sterols and various lipid classes to maintain structural integrity. Similarly, PHR1 and PHR2 are critical proteins in pathogenic *Candida*, whose abundance is modulated by environmental pH. These proteins play essential roles in both morphogenesis and virulence.^58,59^ Furthermore, an SH3 domain-containing protein associated with filamentous growth (GO:0030447) was found to be more abundant in the control group compared to alizarin-treated cells.

## Conclusion

The phytochemical alizarin showed antifungal effects against *C. dubliniensis* primarily by inhibiting the morphological transition and biofilm formation of the fungi. The *G. mellonella* model confirmed this antifungal effect, demonstrating efficacy with negligible modulation of the host’s immune system. Moreover, alizarin was nontoxic for larvae at 16 and 32 μg, highlighting its favorable safety profile as an antifungal agent. Proteomic analysis revealed that alizarin exposure alters the abundance of key proteins involved in cell wall synthesis and energy metabolism, supporting a multifaceted mechanism of action. Notably, alizarin also potentiated the antifungal activity of fluconazole against *C. dubliniensis*, suggesting its promise as an adjunct therapeutic agent. Future research is warranted to fully elucidate the molecular and physiological mechanisms underlying alizarin’s antifungal action. Advanced and integrated omics approaches, including transcriptomics and metabolomics, may provide insights into these complex biological effects. Furthermore, evaluating the potential synergistic effects of alizarin with existing antifungals, particularly in mammalian models of oral candidiasis, could unlock valuable therapeutic strategies for clinically relevant fungal infections. Finally, structural optimization and formulation studies will be essential to improve bioavailability and clinical efficacy, thereby advancing alizarin from a bioactive phytochemical to promising antifungal drug candidate.

## Materials and Methods

### Strains, Media, and Chemicals

All ATCC strains and the clinical isolate (P3) were obtained from the microbial culture collection of the Dental School at ICT-UNESP, São José dos Campos. The clinical isolate of *C. dubliniensis* (P3) was confirmed using the Bruker MALDI Biotyper system (Bruker Daltonics, Bremen, Germany) with a confidence score of 2.3. Sabouraud dextrose agar (SDA), Yeast Nitrogen Base (YNB) and presterilized 96-well cell culture plates were purchased from Kasvi, Brazil. Dimethyl sulfoxide (DMSO), alizarin, RPMI-1640 medium, amphotericin B, and fluconazole were purchased from Sigma-Aldrich (Merck).

### Determination of Minimal Inhibitory Concentration and Minimal Fungicidal Concentration

The minimal inhibitory concentration (MIC) of alizarin, fluconazole, anidulafungin, and amphotericin B (Amp B) was determined using the broth microdilution method according to the Clinical and Laboratory Standards Institute (CLSI) guidelines.^60^ Stock solutions of alizarin (10 mg/mL), Amp B (2 mg/mL), and anidulafungin (2 mg/mL) were prepared in DMSO (100%), while fluconazole (2 mg/mL) was dissolved in sterile distilled water. Given the initially unknown antifungal potential of alizarin, a broad concentration range (1024-1 μg/mL) was first evaluated. However, this approach resulted in solvent-related interference. Therefore, a narrower range of 128–0.25 μg/mL was subsequently selected to minimize solvent effects and ensure reliable results. All *Candida* species were enumerated using a hemocytometer and initially suspended in sterile 0.9% saline. The cell suspension was adjusted to a final density of 4 × 10^3^ cells/mL in RPMI 1640 medium for *Candida* species and 4 × 10^4^ cells/mL for *Aspergillus* species. Presterilized, flat-bottom 96-well microtiter plates were used for the MIC assay. The test compounds were serially 2-fold diluted in RPMI medium to yield final concentrations ranging from 128 to 0.25 μg/mL. Plates were incubated at 37 °C for 48 h. Wells with no visible turbidity were considered to indicate inhibition of growth, and the MIC was recorded accordingly. To determine the minimal fungicidal concentration (MFC), a 10 μL aliquot from each well was plated onto Sabouraud Dextrose Agar (SDA) plates and incubated for 48 h. The MFC/MIC ratio was then calculated to characterize alizarin as fungicidal (MFC/MIC ≤ 4) or fungistatic (MFC/MIC > 4).^37^

### Growth Kinetics Assay

Following MIC and MFC determination, the growth kinetics of alizarin and Amp B against *C. dubliniensis* (ATCC) and the clinical isolate P3 were assessed based on the protocol described by Scorneaux et al.^61^ Drug solutions and cell suspensions were prepared as described above. In sterile 24-well plates, different concentrations of alizarin (MIC, MIC × 5, MIC × 10, and MFC) and the MIC concentration of Amp B were tested. The cell density was maintained at 4 × 10^3^ cells/mL, consistent with MIC assays. Plates were incubated at 37 °C with constant shaking at 150 rpm. At designated time points (0, 2, 4, 6, 24, and 48 h), 10 μL aliquots of the culture were collected and plated onto SDA plates to determine colony-forming units (CFUs). Samples collected at 24 and 48 h were serially diluted in sterile saline prior to plating. After 48 h of incubation, CFUs were counted, and growth curves were constructed accordingly.

### Antibiofilm Activity of Alizarin

The antibiofilm potential of alizarin against biofilm formation and eradication in *C. dubliniensis* was evaluated by quantifying fungal biomass using the crystal violet staining method (Manoharan et al., 2017). Briefly, overnight cultures grown in RPMI medium were adjusted to 1 × 10^6^ cells/mL in sterile PBS. For the biofilm inhibition assay, 200 μL of the standardized cell suspension was dispensed into 96-well plates and incubated under constant shaking for 90 min to allow initial adhesion. Subsequently, nonadherent cells were removed by washing with sterile PBS, and 200 μL of RPMI medium containing different concentrations of alizarin (MIC, MIC × 5, and MIC × 10) was added. The plates were then incubated at 37 °C for 24 h. For the biofilm eradication assay, a separate plate was prepared by adding the standardized cell suspension and allowing biofilm formation for 24 h. After incubation, nonadherent cells were removed by washing with PBS, and 200 μL of RPMI medium supplemented with alizarin (MIC, MIC × 5 and MIC × 10) was added. The plates were further incubated for an additional 24 h at 37 °C. Following incubation, planktonic cells were removed from both assays by repeated washing with PBS, and the remaining biofilms were fixed with 100% ethanol for 10 min. The plates were then stained with 0.1% crystal violet for 40 min and subsequently destained using ethanol. Finally, 100 μL of the destaining solution was transferred to a new plate, and absorbance was measured at 570 nm using a microplate reader (BioTek Synergy). Biomass (%) = T/C*100 and Biomass reduction (%) = [1 – (T/C)] × 100, where *C* represents the absorbance of the control and *T* represents the absorbance of the treated samples. The data presented represent the mean of two independent experiments, each performed with four technical replicates.

### Synergistic Effect of Alizarin with Antifungals

The antifungal synergistic activity of alizarin and common antifungals such as fluconazole, amphotericin B, and anidulafungin was evaluated using a checkerboard microdilution assay following the methodology described by Lima et al.^62^ Reference and clinical strains of *C. dubliniensis* were subcultured on SDA plates and incubated at 37 °C for 24 h. 2-fold serial dilutions of fluconazole (0.5 μg/mL), amphotericin B (0.25 μg/mL), anidulafungin (0.015 μg/mL), and alizarin (16 μg/mL) were prepared in RPMI across eight concentrations, yielding final ranges of 0.5–0.0039 μg/mL, 0.25–0.0020 μg/mL, 0.015–0.00012 μg/mL, and 16–0.125 μg/mL, respectively. The checkerboard assay was performed in 96-well microtiter plates, with Alizarin serially diluted along the *X*-axis and antifungals along the *Y*-axis. Each well was inoculated with a standardized fungal suspension adjusted to a final concentration of 10^4^ CFU/mL in a final volume of 100 μL per well. Plates were incubated at 37 °C for 24 h. Additionally, growth and sterility controls were included, along with single-agent controls to confirm the MIC of each treatment alone. Next, aliquots of 5 μL per well were transferred onto SDA plates agar and incubated for 24 h at 37 °C. The combined MIC was defined as the lowest concentration that completely inhibited microbial growth.

The synergistic effect between alizarin and antifungals was assessed by calculating the fractional inhibitory concentration index (FICI) using the equation

$$FICI=\frac{MIC\;in\;combination}{MIC\;alizarin\;alone}+\frac{MIC\;in\;combination}{MIC\;antifungal\;alone}$$

Synergy was defined as a FICI ≤ 0.5; additive effect as 0.5 < FICI ≤ 1.0; indifference as 1.0 < FICI ≤ 4.0; and antagonism as FICI > 4.0. All experiments were performed in duplicate and repeated independently on two separate occasions.

### Evaluation of In Vivo Antifungal Effects and Immune Modulatory Potential of Alizarin

The in vivo antifungal activity and immunomodulatory potential of alizarin against *C. dubliniensis* were assessed using the *G. mellonella* larvae infection model. Larvae were infected with the clinical isolate (P3) or ATCC strain of *C. dubliniensis*. The experimental protocol was adapted from De Barros et al. (2021).^63^ Healthy *G. mellonella* larvae (weighing 250–300 mg each) were obtained from the Invertebrate Model Research Laboratory, UNESP/ICT. Prior to experimentation, larvae were acclimatized for 24 h at room temperature. *C. dubliniensis* cells and alizarin were suspended in sterile saline. Injections of 10 μL were administered to each larva, with the following groupings: (Amp B: 10 μL of MIC × 1 amphotericin B (positive control), Ali: 10 μL of MIC × 1 alizarin (infected), 2 h Ali: 10 μL of MIC × 1 alizarin injected 2 h post *C. dubliniensis* infection, 24 h Ali: 10 μL of MIC × 1 alizarin injected 24 h prior to *C. dubliniensis* infection, PBS group: 10 μL of sterile saline (uninfected control), Disease control (DC): 10 μL of *C. dubliniensis* cell suspension followed by 10 μL of sterile saline). Larvae survival was monitored over a five-day period, and data were analyzed using Kaplan–Meier survival curves.

To evaluate the immunomodulatory effect of alizarin, a similar experimental setup was used with minor modifications. After 24 h of treatment, larvae were surface sterilized with 70% ethanol. A small incision was made in the lower abdomen using a sterile blade to collect hemolymph aseptically. The hemolymph was immediately mixed with ice-cold sterile insect physiological saline.^63^ The mixture was centrifuged at 1500 × *g* for 5 min at 4 °C, and the pellet was resuspended in sterile PBS to obtain a final hemocyte concentration of 1 × 10^6^ cells/mL. Hemocyte subpopulations were characterized using a FACSLyric flow cytometer (BD Biosciences) based on forward and side scatter properties. A minimum of 10,000 events per sample were recorded and analyzed using BD FACSuite software. In parallel, 10 μL of hemolymph from each larva was plated onto SDA plates to determine the fungal burden by enumerating viable *Candida* colonies, following the protocol described by Rossoni et al.^64^

### Impacts of Alizarin Exposure on Morphological Transition and Proteomic Profile of *C. dubliniensis*

To analyze the relative abundance of proteins in response to alizarin treatment, a shotgun proteomics approach was employed following the protocol described by De Andrade et al.^65^ Briefly, *C. dubliniensis* cells were first cultivated on SDA agar plates, then transferred to YNB medium supplemented with 10% FBS, with or without 16 μg/mL alizarin and incubated at 37 °C for 24 h. The percent hyphal cell formation was determined by counting yeast and hyphal cells using a hemocytometer under light microscopy, and values were expressed as a percentage of hyphal cells relative to the total number of cells counted. Furthermore, cells were collected by centrifugation and lysed using glass bead disruption in a lysis buffer containing 2% CHAPS, 50 mM HEPES, 150 mM NaCl, and a protease inhibitor cocktail (Sigma-Aldrich, USA). The supernatant was then collected, mixed with an equal volume of ice-cold acetone and methanol (4:1), and stored at −80 °C for 2 h to precipitate proteins. After centrifugation at 14,000*g* for 15 min at 4 °C, the resulting pellet was washed several times with ice-cold methanol. Proteins (100 μg) were denatured in 100 μL of 8 M urea, reduced with 5 mM DTT for 25 min at 56 °C, and alkylated with 14 mM IAA for 30 min at room temperature in the dark. The samples were then digested with 0.2 μg of trypsin and incubated at 37 °C for 16 h. Successful protein digestion was confirmed using 12% SDS-PAGE, and trypsin activity was terminated by adding 5% formic acid to a final concentration. Finally, the samples were desalted using C18 membranes (Octadecyl C18-bonded silica—3 M Empore extraction disks) and dried using a vacuum system (miVac, Genevac Ltd.).

### LC–MS/MS Analysis

LC–MS/MS analyses were performed on a Vanquish Neo UPLC system (Thermo Scientific, Waltham, MA, USA) coupled to an Orbitrap Exploris 480 mass spectrometer (Thermo Fisher Scientific, Waltham, MA, USA). A total of 300 ng per sample was injected onto a PepMap Neo precolumn (Thermo Scientific) containing 5 mm of 5 μm C18 beads, and chromatographic separation of the peptides was performed on a PepMap Neo analytical column (Thermo Scientific) with 150 mm of 2 μm C18 beads, using an acetonitrile/0.1% formic acid gradient with steps of 2–45% over 55 min, 45–99% in 0.1 min, and 99% for 5 min, at a flow rate of 300 nL/min. The mass spectrometer was set to operate at 2.1 kV in positive ion mode, with the transfer tube temperature set to 270 °C. It was operated in data-dependent acquisition (DDA) mode using high-energy collision-induced dissociation, with a resolution of 60,000 for MS1 precursors, a normalized AGC target of 300%, a maximum injection time set to auto, and a resolution of 30,000 for MS2 fragments. The scan range was set to a 400–1600 *m*/*z* window and positive charge states of 2–6.

### Proteomics Data Processing

Raw LC–MS/MS data were analyzed using MaxQuant (version 2.0.3.1), with MS/MS spectra searched against the *C. dubliniensis* reference strain database downloaded from UniProt (accessed on October 01, 2024, containing 5829 entries). The search parameters included trypsin/P with up to two missed cleavages, carbamidomethylation of cysteines as a fixed modification, and oxidation of methionine, *N*-terminal acetylation of proteins, and deamidation as variable modifications. The initial tolerances for FTMS and ITMS MS/MS were set at 0.02 and 0.5 Da, respectively. The protein and peptide FDRs were set at 1% with a minimum peptide length of eight amino acids. The variability among biological replicates was assessed by using the Pearson correlation coefficient. A Venn diagram was generated using an online Web server (http://bioinformatics.psb.ugent.be/webtools/Venn/) to distinguish common and unique proteins between alizarin-treated and control groups. Differential protein expression was visualized using a volcano plot, created with the gplot package in the *R* environment. Finally, upregulated proteins in both the control and treatment groups were subjected to gene ontology (GO) analysis using ShinyGO 0.82 (https://bioinformatics.sdstate.edu/go/), with ontology terms specific to *C. dubliniensis*.

### Statistical Analysis

All experiments were performed independently in triplicate, and data are presented as mean ± standard deviation. Statistical significance among groups was evaluated using one-way analysis of variance (ANOVA), followed by Tukey’s post hoc multiple comparisons test. Survival analysis of *G. mellonella* was conducted using Kaplan–Meier survival curves. All statistical analyses were performed using GraphPad Prism (version 9.0 for Windows).
